# Influence of Brain Metastasis on Analgesia-Related Outcomes in Patients with Lung and Breast Cancers Treated with Naldemedine: A Propensity Score-Matched Analysis

**DOI:** 10.3390/jcm12226997

**Published:** 2023-11-09

**Authors:** Aya Hanamoto, Takenao Koseki, Ayaka Utsunomiya, Takuma Ishihara, Takao Tobe, Masashi Kondo, Yuko Kijima, Hiroshi Matsuoka, Tomohiro Mizuno, Takahiro Hayashi, Shigeki Yamada

**Affiliations:** 1 College of Pharmacy, Kinjo Gakuin University, Nagoya 463-8521, Japan; y1871111@kinjo-u.ac.jp (A.H.);; 2 Department of Pharmacotherapeutics and Informatics, Fujita Health University School of Medicine, Toyoake 470-1192, Japantakao.tobe@fujita-hu.ac.jp (T.T.);; 3 Innovative and Clinical Research Promotion Center, Gifu University Hospital, Gifu 501-1194, Japan; 4 Department of Respiratory Medicine, Fujita Health University School of Medicine, Toyoake 470-1192, Japan; 5 Department of Breast Surgery, Fujita Health University School of Medicine, Toyoake 470-1192, Japan; 6 Department of Surgery, Fujita Health University School of Medicine, Toyoake 470-1192, Japan

**Keywords:** naldemedine, brain, opioid, analgesics, lung neoplasm, breast neoplasm

## Abstract

Naldemedine is structurally designed to prevent passage across the blood–brain barrier (BBB), resulting in the attenuation of opioid-induced constipation without interfering with the analgesic effects of opioids. However, the influence of brain metastasis (BM), as one indicator of BBB disruption, on the analgesic effects of opioids in patients treated with naldemedine remains unclear. To examine whether the analgesic effects of opioids following naldemedine treatment are lower in patients with BM than in those without BM, we surveyed inpatients with lung and breast cancers treated with naldemedine at Fujita Health University Hospital between April 2017 and March 2022. Changes in the numeric rating scale (NRS) scores, morphine milligram equivalents (MMEs), and the number of rescues were assessed as analgesia-related outcomes during the first 7 days of naldemedine treatment in patients with or without BM, matched by the propensity score. In total, 172 patients were enrolled. After propensity-score matching, 30 patients with BM and 60 patients without BM were included in the analysis. Changes in NRS scores, MMEs, and the number of rescues did not differ between patients with and without BM. In the linear mixed-effects model, the coefficient of interaction between patients with or without BM and the days for each outcome was not statistically significant. BM does not influence the analgesic effect of opioids in patients with lung and breast cancers treated with naldemedine. Naldemedine may be useful for treating BM.

## 1. Introduction

Opioid analgesics act on the μ-opioid receptor (MOR) in the central nervous system (CNS) to moderate cancer pain. However, MOR activation expressed in the peripheral nervous system by opioid analgesics results in several adverse events, particularly bowel dysfunction, such as opioid-induced constipation (OIC) [1]. OIC is the most frequently reported and persistent adverse event in patients receiving opioid analgesics [2]. Naldemedine, a peripherally acting MOR antagonist (PAMORA) with a high binding affinity for MOR, attenuates OIC by blocking the peripheral MOR activation of opioids without affecting analgesic activity in the CNS [3]. Naldemedine is structurally designed to prevent passage across the blood–brain barrier (BBB) and is also a substrate for p-glycoprotein, resulting in limited entry into the CNS [4]. The BBB restricts the diffusion of specific molecules into the CNS and regulates normal brain function and the homeostasis of CNS through forming a neurovascular unit that includes endothelial cells constituting tight junctions, pericytes, and astrocytes [5,6]. In general, the BBB is disrupted and more permeable in patients with brain metastasis (BM) owing to the loss of tight junctions and increased fenestration of transcytotic vesicles in endothelial cells [6,7]. Naldemedine, a low-molecular-weight compound, may also be transferred to the CNS owing to the loss of tight junctions in patients with BM. Although the specific mechanism remains unknown, high doses of naldemedine were reported to inhibit the analgesic effects of morphine in a rat tail-flick test in a preclinical study [8]. Thus, naldemedine may be transferred to the CNS to reduce the analgesic effects of opioids in patients with clinical cancers and BBB disruption, such as BM [9,10,11].

Osaka et al. reported that naldemedine did not change the numeric rating scale (NRS) scores in a subgroup of patients with BM in two randomized controlled trials of naldemedine for patients with cancer with OIC [12]. However, the NRS score alone as an outcome is limited in assessing the analgesic effects of opioids because the NRS responds to increasing the opioid dosage or adding rapid opioid analgesic rescue to prevent an increase in the NRS. In addition, patient backgrounds may not have been similar in the subgroup analysis because patients were not assigned to assess the differences in NRS scores between those with and without BM. Therefore, further evidence on the influence of naldemedine on the analgesic effects of opioids in patients with cancer, with or without BM, is needed.

BM preferentially originates from lung and breast cancers [13]. To minimize bias across cancer types, patients with lung and breast cancers, who had a higher incidence of BM, were considered suitable for evaluating the effect of naldemedine. Here, we investigate whether three analgesia-related outcomes, i.e., NRS scores, morphine milligram equivalents (MMEs), and the number of rescues, are influenced by naldemedine treatment in patients with lung and breast cancers with BM compared with those without BM.

## 2. Materials and Methods

### 2.1. Study Design and Patient Selection

Japanese inpatients with lung and breast cancers treated with naldemedine at Fujita Health University Hospital between April 2017 and March 2022 were surveyed in this retrospective observational study (ethical approval number: HM22-344). The inclusion criteria were as follows: patients diagnosed with lung or breast cancers and inpatients treated with naldemedine and opioid analgesics during the study periods. The exclusion criteria were as follows: patients treated with naldemedine before hospitalization, those treated with naldemedine within 2 days, those treated with naldemedine without opioid use, and those treated with naldemedine as needed.

Of the 240 patients, 172 were included in this study after excluding 68 patients for the following reasons: 57 patients were treated with naldemedine before hospitalization, 8 patients were treated with naldemedine within 2 days, 2 patients were treated with naldemedine without opioid use, and 1 patient was treated with naldemedine as needed. To investigate whether naldemedine reduced the analgesic effects of opioids in patients with BM, 172 patients were divided into 2 groups: patients with or without BM. After propensity-score matching (PSM), as described in the statistical analysis, patients with BM (n = 30, BM group) and without BM (n = 60, non-BM group) were identified for the analysis.

### 2.2. Data Collection

The data were collected from electronic medical records at Fujita Health University Hospital. The day of the first naldemedine treatment was defined as day 1, and the data regarding NRS scores, MMEs, and the number of rescues from day 0 (1 day before the 1st naldemedine treatment day) to day 7 were collected as analgesia-related outcomes. MMEs were converted to opioid doses according to the conversion factor table by referring to the CDC’s standard conversion factor, clinical guidelines for the pharmacological management of cancer pain in 2020 published by The Japanese Society of Palliative Medicine, and package product labeling of each opioid analgesic in Japan (Supplemental Table S1). The number of rescues included the number of times analgesics were administered orally or rectally and the number of times sustained analgesics were administered rapidly. To adjust for factors that may have influenced cancer pain, we collected data regarding the presence of bone metastasis and radiotherapy for pain relief. Data in relation to the coexistence of hypertension, stroke, schizophrenia, depression, and epilepsy were also collected as those condition may alter BBB function and affected its permeability [14].

### 2.3. Statistical Analysis

Patient characteristics are presented as medians and interquartile ranges for continuous variables and counts and proportions for categorical variables. To ensure comparability between the BM and non-BM groups, a 1:2 PSM was performed. Propensity scores were estimated using logistic regression models with the group as the objective variable and age; sex; height; body weight; body mass index; cancer type; stage (I–II, III, IV); first occurrence or recurrence of cancer; presence of bone metastasis; radiotherapy for pain relief; coexistence of hypertension, stroke, schizophrenia, depression, or epilepsy; NRS scores; MMEs; and the number of rescues for day 0 as explanatory variables. Caliper values were set using the SD/4 criterion for linearly predicted values from the logistic regression model. Multiple imputation methods were used because missing values were identified as covariates. The least square (LS) means of the outcomes were calculated for each group on each day, and comparisons were conducted between the groups. A linear mixed-effects model was used to evaluate differences over time in the effect of naldemedine on NRS scores, MMEs, and the number of rescues during the first 7 days of naldemedine treatment (changes from day 0) between the BM and non-BM groups. Groups, days, and their interaction terms were included in the model as fixed effects, and a randomly varying intercept between patients was applied. If the interaction between the group and days was statistically significant, the BM was used to modify the effect of naldemedine. A two-sided significance level of 5% was considered. R version 4.2.2 was used for all analyses (www.r-project.org).

## 3. Results

### 3.1. Patient Characteristics

**Figure 1:**
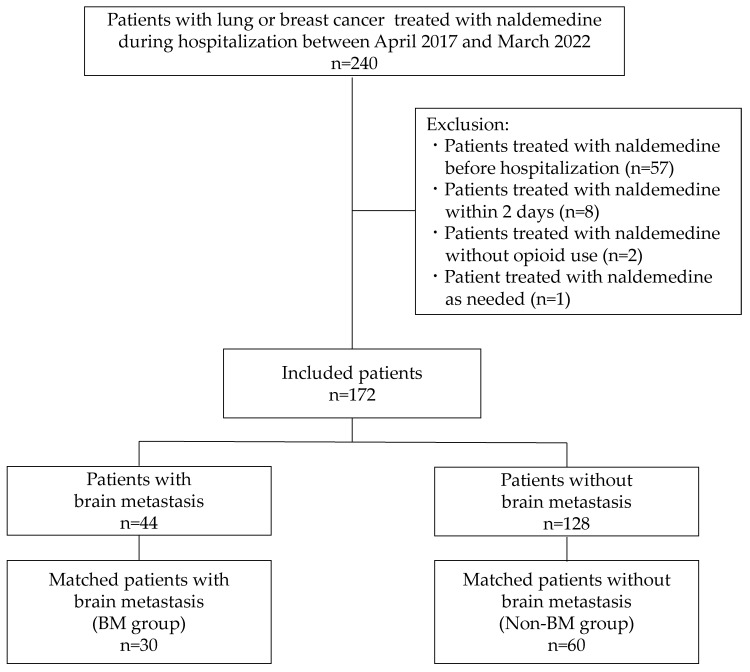
Patient selection flowchart. BM, patients with brain metastasis; non-BM, patients without brain metastasis.

This study included 172 patients with lung and breast cancers treated with naldemedine during hospitalization, and 90 patients were matched as the analysis population (Figure 1). The baseline characteristics of the study patients are shown in Table 1. Patients with or without BM were divided into BM and non-BM groups. Before PSM, there was a bias between the BM and non-BM groups in terms of several variables, such as age, presence of radiotherapy, and coexistence of schizophrenia. After PSM, the median age was 69.0 years in both groups. In the BM and non-BM groups, 66.7% and 61.7% were male, 83.3% and 81.7% had lung cancer, and 80.0% and 73.3% had stage IV cancer, respectively. On day 0, the median NRS scores were 4.0 and 4.0, the median MMEs were 30.0 and 22.5, and the median number of rescues were 1.0 and 1.0 in the BM and non-BM groups, respectively. In the analysis cohort after PSM, the BM and non-BM groups had comparable characteristics and baseline analgesia-related outcomes.

### 3.2. Changes in Analgesia-Related Outcomes

**Figure 2:**
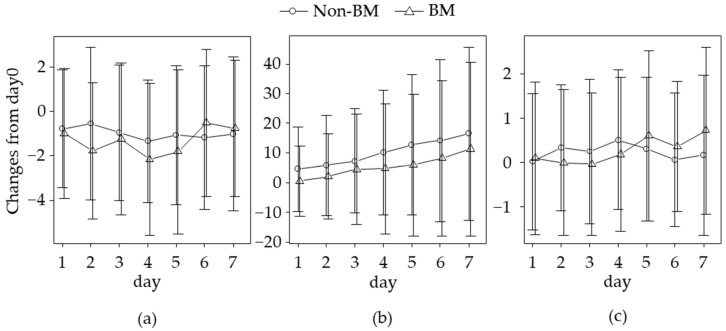
Changes in numeric rating scale scores (**a**), morphine milligram equivalents (**b**), and the number of rescues (**c**) during the first 7 days of naldemedine treatment. Values indicate the mean ± standard deviation. BM, patients with brain metastasis; MMEs, morphine milligram equivalents; NRS, numeric rating scale; non-BM, patients without brain metastasis.

Changes in the NRS scores, MMEs, and number of rescues from day 0 and LS means on days 3 and 7 during the first 7 days of naldemedine treatment are shown in Figure 2 and Table 2, respectively. NRS scores did not change over the 7 days, and the LS mean difference (95% confidence interval (CI)) between the BM and non-BM groups on days 3 and 7 was −0.63 (95% CI, [−1.84, 0.58]; *p* = 0.305) and −0.30 (95% CI, [−1.67, 0.11]; *p* = 0.666), respectively. Changes in MMEs increased day by day in both groups, and the LS mean difference between the BM and non-BM groups on days 3 and 7 was −4.32 (95% CI, [−12.97, 4.33]; *p* = 0.323) and −5.57 (95% CI, [−14.71, 3.57]; *p* = 0.230), respectively. The number of rescues did not alter over the 7 days, and the LS mean difference between the BM and non-BM groups on days 3 and 7 was −0.13 (95% CI, [−0.72, 0.45]; *p* = 0.651) and 0.15 (95% CI, [−0.53, 0.82]; *p* = 0.668), respectively.

### 3.3. Association of BM and Outcome Changes over Time

The coefficient of the group, days, interaction between the group and days for the changes from day 0 during the first 7 days of naldemedine treatment in terms of NRS scores, MMEs, and the number of rescues by the linear mixed-effect model are shown in Table 3. The coefficient of interaction between group and days for each outcome was not statistically significant (coefficient [95% CI]: NRS, 0.08 [−0.12, 0.29], *p* = 0.435; MMEs, −0.31 [−1.26, 0.64], *p* = 0.519; number of rescues, 0.07 [−0.04, 0.18], *p* = 0.195). Thus, the changes in each outcome over time did not differ between the BM and non-BM groups.

## 4. Discussion

Changes in analgesia-related outcomes, such as NRS scores, MMEs, and the number of rescues in patients with lung and breast cancers with or without BM treated with naldemedine, were analyzed in the PSM population in this study. Patients with BM did not show higher MMEs, number of rescues, or NRS scores with naldemedine treatment than those without BM.

Osaka et al. reported that naldemedine had no effect on NRS score changes in patients with BM in a subgroup post hoc analysis of two randomized studies [12]. In addition to this report, our finding that naldemedine does not affect MMEs and the number of rescues or NRS scores in patients with BM suggests that naldemedine has little influence on the analgesic effects of opioids in patients with or without BM, even when evaluated complexly by considering various analgesia-related outcomes.

Naldemedine is a derivative of naltrexone with a carbamoyl group that increases the polarity and molecular weight, resulting in an inability to cross the BBB [15]. However, in a preclinical study, a high dose of naldemedine inhibited the analgesic effect of morphine in a rat tail-flick test [8], suggesting that naldemedine had the potential to reduce the analgesic effect of morphine in humans. Several PAMORAs, such as methylnaltrexone, naloxegol, and naldemedine, have been used to manage OIC [16]. BBB penetration was lower with naldemedine than with methylnaltrexone, the first PAMORAs approved by the US Food and Drug Administration, based on pre-clinical testing (anti-analgesic test and small intestinal transit test) [8]. Methylnaltrexone also did not influence the pain score in patients with BM in a post hoc analysis of three randomized clinical studies [17]. Few studies have reported the analgesic effects of naloxegol on opioids in patients with BM. Thus, at clinically used doses, naldemedine, which does not reduce the analgesic effects of opioids, may be an option for OIC treatment in patients with BM.

Our study had several limitations. First, the target cancer types were limited to patients with lung and breast cancers who frequently had BM. The structural integrity and permeability of the BBB were heterogeneous between metastatic lesions and different types of primary tumors [18]. Thus, to minimize the ratio of BM/non-BM patients and BBB heterogeneity due to the inclusion of various cancer types, the target patients were limited to lung and breast cancer patients; however, it is unclear whether the results would be similar for other cancer types. Second, the long-term influence of naldemedine treatment beyond the first 7 days on the analgesic effect of opioids in patients with BM remains unknown. Naldemedine may be used for a long period as long as opioids are used, but a long-term evaluation was difficult because target inpatients were hospitalized in acute hospital wards, and analgesia-related outcomes could not be tracked owing to discharge or other reasons. Third, the reactivity of pain may possibly vary depending on the region of BM; however, we were unable to assess the differences in the region of BM. Finally, this was a single-site observational study, and the results are not as generalizable as those of a multisite evaluation.

## 5. Conclusions

In conclusion, analgesia-related outcomes in patients with lung and breast cancers with BM were not influenced by naldemedine treatment. Although further investigations are needed, the present results provide evidence that naldemedine may be used for OIC in patients with BM without compromising the analgesic effects of opioids.

## Figures and Tables

**Table 1:** Baseline characteristics of patients.

	Before PSM			After PSM		
	BM n = 44	Non-BM n = 128	SMD	BM n = 30	Non-BM n = 60	SMD
Age, years	73.0 (63.0, 80.0)	68.5 (60.0, 71.3)	0.396	69.0 (62.3, 71.0)	69.0 (59.0, 74.0)	0.031
Sex						
Male	29 (65.9)	79 (61.7)	0.087	20 (66.7)	37 (61.7)	0.104
Female	15 (34.1)	49 (38.3)		10 (33.3)	23 (38.3)	
BMI, kg/m^2^	21.1 (18.0, 22.5)	20.4 (18.0, 23.2)	0.065	21.6 (18.2, 22.3)	21.0 (18.7, 23.1)	0.053
Cancer type						
Lung cancer	38 (86.4)	104 (81.2)	0.139	25 (83.3)	49 (81.7)	0.044
Breast cancer	6 (13.6)	24 (18.8)		5 (16.7)	11 (18.3)	
Stage						
I–II	2 (4.5)	17 (13.3)	0.377	2 (6.7)	5 (8.3)	0.160
III	5 (11.4)	22 (17.2)		4 (13.3)	11 (18.3)	
IV	37 (84.1)	89 (69.5)		24 (80.0)	44 (73.3)	
Recurrent cancer	7 (15.9)	38 (29.7)	0.333	6 (20.0)	13 (21.7)	0.041
Bone metastasis	28 (63.6)	65 (50.8)	0.262	13 (43.3)	23 (38.3)	0.102
RT for pain relief	20 (45.5)	94 (73.4)	0.595	15 (50.0)	32 (53.3)	0.067
Coexisting disease						
Hypertension	15 (34.1)	49 (38.3)	0.087	11 (36.7)	23 (38.3)	0.034
Stroke	6 (13.6)	20 (15.6)	0.056	4 (13.3)	7 (11.7)	0.050
Schizophrenia	4 (9.1)	0 (0.0)	0.447	0 (0.0)	0 (0.0)	<0.001
Depression	3 (6.8)	4 (3.1)	0.171	0 (0.0)	1 (1.7)	0.184
Epilepsy	3 (6.8)	2 (1.6)	0.265	0 (0.0)	1 (1.7)	0.184
Day 0 outcome values						
NRS	4.0 (1.3, 6.0)	4.0 (1.0, 6.0)	0.015	4.0 (1.0, 6.0)	4.0 (2.0, 6.0)	0.053
MMEs	30.0 (20.0, 45.0)	22.5 (15.0, 60.0)	0.106	30.0 (18.8, 45.0)	22.5 (15.0, 50.0)	0.106
Number of rescues	1.0 (0.0, 2.0)	1.0 (0.0, 2.0)	0.114	1.0 (0.0, 2.0)	1.0 (0.0, 1.0)	0.140

Data indicate median (25th–75th percentiles) or number (%). BM, patients with brain metastasis; BMI, body mass index; MMEs, morphine milligram equivalents; non-BM, patients without brain metastasis; NRS, numeric rating scale; PSM, propensity-score matching; RT, radiotherapy; SMD, standardized mean difference.

**Table 2:** Least square means of changes from day 0 for numeric rating scale score, morphine milligram equivalents, and the number of rescues at days 3 and 7 after starting naldemedine treatment in the propensity-score-matched cohort.

	LS Mean Changes in BM (95% CI)	LS Mean Changes in Non-BM (95% CI)	Difference: BM Minus Non-BM (95% CI)	*p*-Value
NRS				
Day 3	−1.44 (−2.45, −0.43)	−0.81 (−1.48, −0.14)	−0.63 (−1.84, 0.58)	0.305
Day 7	−1.29 (−2.42, −0.15)	−0.99 (−1.76, −0.22)	−0.30 (−1.67, 0.11)	0.666
MMEs				
Day 3	3.78 (−3.30, 10.90)	8.10 (3.14, 13.10)	−4.32 (−12.97, 4.33)	0.323
Day 7	10.3 (2.81, 17.80)	15.9 (10.63, 21.10)	−5.57 (−14.71, 3.57)	0.230
Number of rescues				
Day 3	0.13 (−0.34, 0.61)	0.26 (−0.08, 0.60)	−0.13 (−0.72, 0.45)	0.651
Day 7	0.52 (−0.03, 1.07)	0.37 (−0.02, 0.76)	0.15 (−0.53, 0.82)	0.668

BM, patients with brain metastasis; CI, confidence interval; LS, least square; MMEs, morphine milligram equivalents; non-BM, patients without brain metastasis; NRS, numeric rating scale.

**Table 3:** Association of numeric rating scale scores, morphine milligram equivalents, and the number of rescues for 7 days after starting naldemedine treatment with BM and days in the propensity-score-matched cohort.

	Coefficient	95% CI	*p*-Value
NRS			
BM (vs. non-BM)	−0.88	−2.29, 0.54	0.229
Days	−0.04	−0.16, 0.07	0.467
Interaction of BM and days	0.08	−0.12, 0.29	0.435
MMEs			
BM (vs. non-BM)	−3.38	−12.57, 5.81	0.400
Days	1.94	1.40, 2.48	<0.001
Interaction of BM and ays	−0.31	−1.26, 0.64	0.519
Number of rescues			
BM (vs. non-BM)	−0.34	−1.03, 0.34	0.331
Days	0.03	−0.03, 0.09	0.382
Interaction of BM and days	0.07	−0.04, 0.18	0.195

BM, patients with brain metastasis; CI, confidence interval; MMEs, morphine milligram equivalents; non-BM, patients without brain metastasis; NRS, numeric rating scale.

## Data Availability

Third-party provision of the data was not allowed because it was not reviewed or approved by the Ethics Committee.

## Supplementary Materials

The following supporting information can be downloaded at: https://www.mdpi.com/article/10.3390/jcm12226997/s1, Table S1: Morphine milligram equivalents conversion factor list.

## Abbreviations

BBB	blood–brain barrier
BM	brain metastasis
CI	confidence interval
CNS	central nervous system
LS	least square
MMEs	morphine milligram equivalents
MOR	μ-opioid receptor
NRS	numeric rating scale
OIC	opioid-induced constipation
PAMORA	peripherally acting μ-opioid receptor antagonist
PSM	propensity-score matching
