# Supplementary material for: Influence of Brain Metastasis on Analgesia-Related Outcomes in Patients with Lung and Breast Cancers Treated with Naldemedine: A Propensity Score-Matched Analysis
Source: J Clin Med. 2023 Nov 9;12(22):6997. doi: 10.3390/jcm12226997 (PMC10672656; doi:10.3390/jcm12226997)
Supplement: Supplementary file 1 [file jcm-12-06997-s001.zip › jcm-2664708-supplementary.pdf]

Supplemental Table S1. Morphine milligram equivalents conversion factor list

	Conversion factor
Morphine Sulfate Hydrate (po)	1.0
Morphine Hydrochloride Hydrate (po)	1.0
Morphine Hydrochloride Hydrate (iv)	2.0
Oxycodone Hydrochloride Hydrate (po)	1.5
Oxycodone Hydrochloride Hydrate (iv)	2.0
Hydromorphone Hydrochloride (po)	5.0
Hydromorphone Hydrochloride (iv)	15.0
Tapentadol Hydrochloride (po)	0.3
Tramadol Hydrochloride (po)	0.2
Tramadol Hydrochloride・Acetaminophen (po)	0.2
Codeine Phosphate Hydrate (po)	0.15
Fentanyl Citrate transdermal	30.0
Fentanyl Citrate (iv)	100.0

iv, intravenous administration; po, per OS.
